# Supplementary material for: scPRINT: pre-training on 50 million cells allows robust gene network predictions
Source: Nat Commun. 2025 Apr 16;16:3607. doi: 10.1038/s41467-025-58699-1 (PMC12003772; doi:10.1038/s41467-025-58699-1)
Supplement: Supplementary file 1 — Supplementary Information [file 41467_2025_58699_MOESM1_ESM.pdf]

Supplementary Information: What can 50 million cells tell us about gene networks?

Table S1: List of novelties in scPRINT and comparison to scGPT and scFoundation

features	scPRINT	scGPT	scFoundation	Geneformer v2
classification pretraining	v	x	x	x
hierarchical classification	v	x	x	x
denoising pretraining	v	x	v	x
masking pretraining	v	v	v	v
MVC pretraining	v	v	x	x
AE pretraining	v	x	x	x
large cell count GN inference	v	x	x	x
zero-shot classification	v	x	x	x
zero-shot batch correction	v	x	x	x
zero-shot denoising	v	x	x	x
genome-wide GN inference	v	x	x	x
large input context	x	x	v	x
raw count encoding	v	x	v	x
very large model	v	x	x	x
pretraining strategy and dataset	v	x	x	x
low GPU/hours implementation	v	x	x	x
weighted random sampling	v	x	x	x
protein encoding	v	x	x	x
cross-species abilities	v	x	x	x
gene location encoding	v	x	x	x
genome-wide input context	x	x	v	x
xtrimogene architecture	x	x	v	x
train / validate / test strategies	v	x	x	x
flashattention2	v	x	x	x

Comparison of the features and novelties from scPRINT compared to 2 similar published state-of-the-art methods: scGPT and scFoundation.

Table S2: model comparison

**model name**	**model size**	**training time (hours)**	**training hardware**	**num cells**	**num leaf cell type**	**dimension (d)**	**layers**	**heads**	**token input size**	**num species**	**training**	**attention**
**scPRINT-small**	7M	24	1xA100	41M (91M before QC)							denoising (60%) + classification + bottleneck	flashattention2
**Geneformer v2**	? (~50M)	72	12xV100	30M	?	256	6	4	2,048	1	masked (15%)	normal
**scPRINT-medium**	20M	72	1xA100	41M (91M before QC)	540	256	8	4	2,200	2	denoising (60%) + classification + bottleneck	flashattention2
**scGPT**	100M	?	?	33M	?	512	12	8	1,200	1	masked (15%)	flashattention1
**scFoundation**	100M	?	?	50M	?	768	12+12	12+8	20,000	1	masked (30%) + denoising	xtrimogene
**scPRINT**	90M	96	4xA100	41M (91M before QC)	540	512	16	8	2,200	2	denoising (60%) + classification + bottleneck	flashattention2
**GPT2-small**	117M	?	?	300B tokens (~150M cells)	x	768	12	12	1200	x	masked (15%)	normal
**UCE**	650M	960	24xA100	36M	(~1000?) likely <500	1280	33	20	1024	5	masked (20%)	normal
**cellFM**	700M	?	32xAscend910 NPUs	100M	?	1536	40	48	4096	1	masked (20%)	normal + LORA
**scPRINT-vlarge**	700M	168	24xA100	41M (91M before QC)	540	1280	20	10	2,200	2	denoising (60%) + classification + bottleneck	flashattention2

Table comparing different model sizes and architectures. Comparing scPRINT to other state-of-the-art methods, as well as GPT2-small and GPT3-large models

Table S3: Ablation study and impact on performance across tasks

id	description	denoise/reco2full_vs_noisy2full	emb_lung/ct_class	emb_lung/scib	emb_panc/ct_class	emb_panc/scib	reconstruction loss	classification accuracy	denoising loss	epoch
**or46096v**	small	0.34	0.31	0.47	0.11	0.41	1.31	0.4	1.16	24
**ghqf2hym**	medium	0.12	0.58	0.55	0.52	0.51	1.25	0.33	1.125	27
**7asy8qpn**	large	0.18	0.69	0.56	0.52	0.50	1.23	0.76	1.109	21
**24chcp2e**	medium-nofreeze	0.15	0.45	0.54	0.52	0.53	1.25	0.33	1.115	23
**6o76ew23**	medium-2-heads	0.10	0.49	0.55	0.40	0.53	1.25	0.33	1.124	26
**lsr3pvnf**	medium-MSE	0.21	0.61	0.56	0.51	0.49	1.26	0.33	6.3 (diff)	29
**muwj73gx**	medium-MVC	0.21	0.51	0.55	0.40	0.47	1.29	0.3	1.132	37
**n8jypo8z**	medium-noPE	0.09	0.71	0.56	0.35	0.46	1.27	0.33	1.31	23
**q0fzpj5g**	medium-no-random-weighted	0.17	0.51	0.53	0.19	0.48	1.26	0.26	1.118	27
**f5e4qfkr**	medium-MLM	0.04	0.53	0.54	0.39	0.46	1.26	0.35	0.999	23

The table shows the results of the ablation study on denoising, embedding with batch correction, and cell-type classification tasks. Results are displayed for the medium-size scPRINT model. Top to bottom: *small, medium, large:* regular models of various sizes. *medium-nofreeze:* a model trained without freezing gene embedding during pre-training. *medium-2-heads:* a model trained with only two heads per layer instead of 4. *medium-MSE*: a model with Mean Squared Error instead of the ZINB loss. *medium-MVC:* a model trained with scGPT's MVC methodology for the creation of the cell embedding. medium-noPE: a model trained without positional encoding for the gene's location. medium-no-random-weighted: a model trained without weighted random sampling. *medium-MLM*: a model trained with masked language modeling instead of denoising.

Table S4: Computational speed of various GN inference methods

model	speed for 1000 cells	speed for a dataset of 12 cell types	scale to #cells	scale to #genes
**DeepSEM**	10mn	2 hours	linear	quadratic
**GENIE3**	50mn	10 hours	quadratic	quadratic
**GENIE3 (100 trees)**	4mn	1 hour	quadratic	quadratic
**Geneformer v2**	1mn	15mn	linear	linear
**scGPT**	1mn	15mn	linear	linear
**scPRINT**	1mn	15mn	linear	linear

The computational speed of running various gene network inference methods on a set of 4000 genes and 1000 cells. It is showing that transformer-based models are far faster than previous methods, owing to their clever use of the GPU and pre-training.

Table S5: Performance of GN inference methods on the Sergio simulated scRNAseq dataset

model	EPR	AUPRC	TF_targ	TF_enr
**DeepSEM**	0.92601	0.00101	0	FALSE
**GENIE3**	0.94497	0.00193	5.2	TRUE
**Geneformer v2**	0.699	0.00409	0	TRUE
**scGPT**	0.6167	0.00278	10.5	TRUE
**scPRINT**	1.836	0.00861	13.15	FALSE

We generate a Sergio simulated scRNAseq dataset of 1000 cells for 800 genes from the RegNetwork ground truth network. We here showcase the ability of each model to recover the RegNetwork ground truth from this dataset. It shows how only scPRINT can recover some of RegNetwork's connections.

Table S6: Comparison scPRINT model size on performance across tasks and GN inference abilities

id	description	denoise/reco2full_vs_noisy2full	emb_lung/ct_class	emb_lung/scib	emb_panc/ct_class	emb_panc/scib	grn_gwps/auprc	grn_gwps/auprc_omni	grn_gwps/auprc_self	grn_gwps/epr	grn_gwps/epr_omni	grn_gwps/epr_self
**or46096v**	small	0.34	0.31	0.47	0.11	0.41	0.03804	0.038	0.039	4.375	4.375	4.420
**ghqf2hym**	medium	0.12	0.58	0.55	0.52	0.51	0.03183	0.032	0.035	3.239	3.040	3.514
**7asy8qpn**	large	0.18	0.69	0.56	0.52	0.50	0.03407	0.035	0.038	3.466	3.595	4.126

id	grn_omni/auprc	grn_omni/auprc_class	grn_omni/epr	grn_omni/epr_class	grn_omni/tf_enr	grn_omni/tf_enr_class	grn_omni/tf_targ_enr	grn_omni/tf_targ_enr_class	grn_sroy/auprc	grn_sroy/auprc_omni	grn_sroy/auprc_self	grn_sroy/epr	grn_sroy/epr_omni	grn_sroy/epr_self
**or46096v**	0.00137	0.00134	2.042	2.020	2	2	9	4	0.099	0.099	0.106	1.303	1.303	1.538
**ghqf2hym**	0.00148	0.00142	2.111	2.293	1	1	30	21	0.100	0.089	0.088	1.524	0.726	0.540
**7asy8qpn**	0.00139	0.00142	2.021	3.257	6	6	32	30	0.100	0.102	0.108	0.589	0.985	1.322

id	mean epr	mean auprc	mean ct_class	mean scib	total weigthed mean	reconstruction loss	classification accuracy	denoising loss	epoch
**or46096v**	2.429	0.00459272619	0.21	0.44	**0.8902439048**	1.31	0.4	1.16	24
**ghqf2hym**	1.992	0.004071803571	0.55	0.53	**1.023048607**	1.25	0.33	1.125	27
**7asy8qpn**	2.252	0.00436602381	0.60	0.53	**1.096761024**	1.23	0.76	1.109	21

here the comparison between scPRINT small (7M params), scPRINT medium (20M params) and scPRINT large (90M) params. We compare across both almost all metrics presented in results section.
While scPRINT small attains great performances on some gene network inference tasks and on the denoising we see that the overall best model remains scPRINT large

Table S7: overlap of different GN ground truths

comparison	precision	recall	random precision
MCalla et al. vs Omnipath	0.0520	0.0074	0.00154
MCalla et al. - T vs Omnipath	0.0155	0.0022	0.00154
gwps vs Omnipath	0.0015	0.0219	0.00129
gwps -T vs Omnipath	0.0030	0.0426	0.00129

Comparison of the overlap, expressed as precision and recall, of the three different ground truth networks used: MCalla, Omnipath, and gwps.

Table S8: Omnipath benchmark results on the genome-wide perturb-seq dataset

tool	EPR	AUPRC	TF target enr.	TF_enr	TF_only	ct_pred	RAND precision
**DeepSEM**	4.1	0.00192	21.4	FALSE	FALSE	FALSE	0.001633
**GENIE3**	4.7	0.00188	17.9	TRUE	FALSE	FALSE	0.00163
**Geneformer v2**	0.2	0.001796	5.9	FALSE	FALSE	FALSE	0.001528
**scGPT**	1.0	0.00208	14.0	TRUE	FALSE	FALSE	0.00163
**scPRINT**	2.8	0.00170	8.6	TRUE	FALSE	FALSE	0.00161
**scPRINT (omnipath's heads)**	4.7	0.00189	3.4	TRUE	FALSE	FALSE	0.00161
**scPRINT (gwps' heads)**	1.6	0.00190	5.0	TRUE	FALSE	FALSE	0.00161

Omnipath network overlap (EPR, AUPRC), as well as transcription factor enrichment, TF target enrichment, and cell type marker enrichment for gene networks generated by the different tools on the genome-wide perturb seq K562 cells at steady state (no perturbations)

Table S9: Omnipath benchmark results on the MCalla et al. datasets

tool	dataset	EPR	AUPRC	TF target enr.	TF enr.	cell type enr.
**DeepSEM**	**Han et. al.**	5.54	0.00029	18.9	FALSE	FALSE
**DeepSEM**	**Yan et. al.**	0.97	-0.00002	7.5	FALSE	FALSE
**GENIE3**	**Han et. al.**	1.51	0.00016	11.3	FALSE	TRUE
**GENIE3**	**Yan et. al.**	1.74	0.00020	0.0	FALSE	TRUE
**Geneformer**	**Han et. al.**	1.63	0.00010	11.3	FALSE	FALSE
**Geneformer**	**Yan et. al.**	1.99	0.00011	20.0	FALSE	FALSE
**scGPT**	**Han et. al.**	0.89	0.00016	17.0	TRUE	FALSE
**scGPT**	**Yan et. al.**	0.16	0.00007	20.0	FALSE	FALSE
**scPRINT**	**Han et. al.**	2.03	0.00019	23.6	TRUE	FALSE
**scPRINT**	**Yan et. al.**	1.76	0.00026	31.1	FALSE	TRUE
**scPRINT (omnipath's heads)**	**Han et. al.**	5.12	0.00004	3.6	TRUE	FALSE
**scPRINT (omnipath's heads)**	**Yan et. al.**	3.35	0.00019	13.3	FALSE	TRUE
**scPRINT (Han et. al.'s heads)**	**Han et. al.**	0.94	0.00030	30.9	TRUE	TRUE
**scPRINT (Han et. al.'s heads)**	**Yan et. al.**	0.57	-0.00004	6.7	TRUE	TRUE

Omnipath network overlap (EPR, AUPRC), as well as transcription factor enrichment, TF target enrichment, and cell type marker enrichment for gene networks generated by the different tools on the 2 human embryonic stem cell datasets used in scPRINT outperforms GENIE3 and scGPT on cell type-specific ground truths.

Table S10: Denoising results per datasets

tools	denoising (+%) correlation. gNNpgpo6gATjuxTE7CCp	denoising (+%) correlation. R4ZHoQegxXdSFNFY5LGe	denoising (+%) correlation (REIyQZE6OMZm1S3W2Dxi)	denoising (+%) correlation (low cell count: 30). gNNpgpo6gATjuxTE7CCp	denoising (+%) correlation (low cell count: 30). R4ZHoQegxXdSFNFY5LGe	denoising (+%) correlation (low cell count: 30) (REIyQZE6OMZm1S3W2Dxi)	average denoising (+%) correlation	average denoising (+%) correlation (rare cell type)
**untrained scPRINT**	-16.0	X	X	-16.0	X	X	-16.0	-16.0
**scPRINT**	19.1	33.9	17.1	22.5	26.6	16.6	23.4	21.9
**KNNsmoothing2**	21.0	34.9	21.6	17.0	32.0	13.4	25.8	20.8
**magic**	29.3	34.6	22.7	16.8	24.4	4.6	28.9	15.3
**magic (low cell dataset)**	X	X	X	11.3	14.0	13.0	X	12.8

This table shows the detail of the denoising results for each of the three datasets for scPRINT-large, KNNsmoothing2, MAGIC, and MAGIC run on only the small cell type cluster. “Random scPRINT model” is the performance of an untrained scPRINT model.

Table S11: highlighted b-cell cluster genes in the BPH study

gene	link	in cancer	in b cell	analysis
**MBNL2**	https://www.nature.com/articles/s41467-023-44126-w	prostate cancer	high expr in immune tissues	
**MAGOH**	https://www.ncbi.nlm.nih.gov/pmc/articles/PMC9738831/	cancer	high expr in immune tissues	
**RANBP2**	https://www.nature.com/articles/leu2012286, https://www.genecards.org/cgi-bin/carddisp.pl?gene=RANBP2	B-cell lymphoma	b cell validated	
**CLIC4**	https://www.nature.com/articles/s41420-022-01003-7	prostate cancer	high expr in immune tissues	
**BAG5**	https://www.ncbi.nlm.nih.gov/pmc/articles/PMC3598994/	prostate cancer	b cell in cancer	
**NR4A1**	https://www.ncbi.nlm.nih.gov/pmc/articles/PMC9424640/ https://www.ncbi.nlm.nih.gov/pmc/articles/PMC8081071/	prostate cancer	b cell validated	
**BAZ2A**	https://www.nature.com/articles/s41598-024-56073-7	prostate cancer	b cell validated	
**ZBTB16**	https://www.pnas.org/doi/full/10.1073/pnas.0703872104 https://www.ncbi.nlm.nih.gov/pmc/articles/PMC5642638/	tumor suppressor in prostate cancer	b cell validated	BPH B-cell to normal B-cell diff. expr.
**TAP1**	https://bmccancer.biomedcentral.com/articles/10.1186/s12885-023-10527-9 https://www.ncbi.nlm.nih.gov/pmc/articles/PMC5674960/	cancer	b cell validated	
**TAS2R19**	https://v19.proteinatlas.org/ENSG00000212124-TAS2R19/tissue/B-cells		b cell validated	
**PRDM7**	https://pubmed.ncbi.nlm.nih.gov/27129774/	cancer		
**TSEN54**	https://www.ncbi.nlm.nih.gov/pmc/articles/PMC10120902/	cancer	b cell in cancer	
**EHMT2**	https://www.frontiersin.org/journals/immunology/articles/10.3389/fimmu.2017.00429/full		b cell validated	
**ERICH6B**	https://platform.opentargets.org/target/ENSG00000163645/associations	cancer		
**IL10RB**	https://pubmed.ncbi.nlm.nih.gov/37144812/	cancer	b cell in cancer	BPH B-cell to normal B-cell diff. expr. post denoising

Table of the highlighted genes in the differential expression analysis in BPH vs normal B-cells together with their annotation on their relation to cancer and to b-cells, with sources.

Table S12: hub and differential hub genes in the fibroblast GN of the BPH study

TOP 15 hubs in BPH fibroblasts GN	TOP 15 hubs in normal fibroblasts GN	TOP 15 differential hubs in BPH fibroblasts vs normal	TOP 15 eigenvector_centrality differential hubs in BPH fibroblasts vs normal
HSPA1A	S100A6	HLA-A	CD99
MT2A	TGIF2-RAB5IF	MT2A	HLA-A
CREM	MIF	ATP6V0C	HSPA1A
TGIF2-RAB5IF	DNAJB9	DEFA1	LUM
HSPE1	IGFBP7	EIF4A1	ATP6V0C
CALD1	APOD	HSPA1A	CD99
SPOCK3	BRME1	LUM	EIF4A1
HLA-A	SPARCL1	SPOCK3	PAGE4
SPARCL1	TIMP1	nan-99	RYR2
RBP1	DCN	CD99	SERPINF1
C1S	C1S	CPE	C1R
BRME1-1	MGP	THBS1	COL6A2
FABP4	nan-270	LGALS1	HNRNPA0
nan-99	SLC25A6	PYDC2	SERPING1
LUM	BLOC1S5-TXNDC5	SERPING1	SERPINA3

List of the Top-15 elements in different GN analyses. Genes in yellow in the last columns are the new ones found with eigenvector centrality compared to the 3rd columns.

Table S13: number of elements predicted per class

ethnicity	21
sex	2
organism	2
cell type	424
disease	62
assay	26

Number of labels predicted by the model for each class. We use hierarchical classification for cell type, disease, assay, and ethnicity.

Supplementary figures

FIG S1: visualization of human gene embedding from ESM2

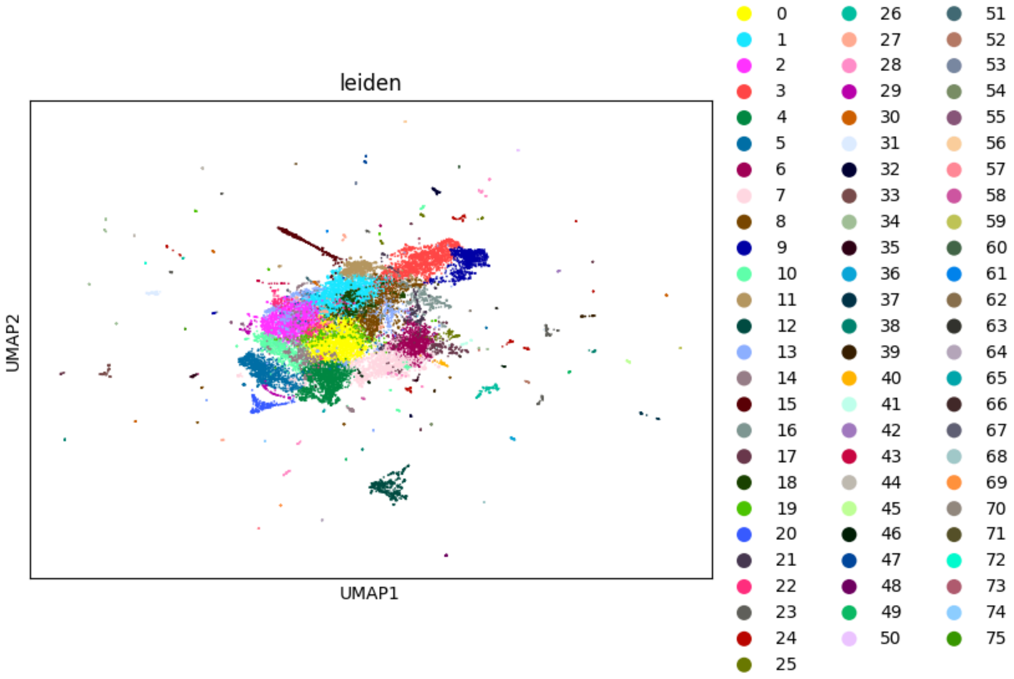

Umap of the ESM2 protein embeddings for the most common protein of all protein-coding genes in Ensembl. The PCA variance ratio is 0.856 for the top 50 principal components. We color it using the Louvain clustering of the embedding.

FIG S2: Gene network inference comparison with Omnipath per datasets

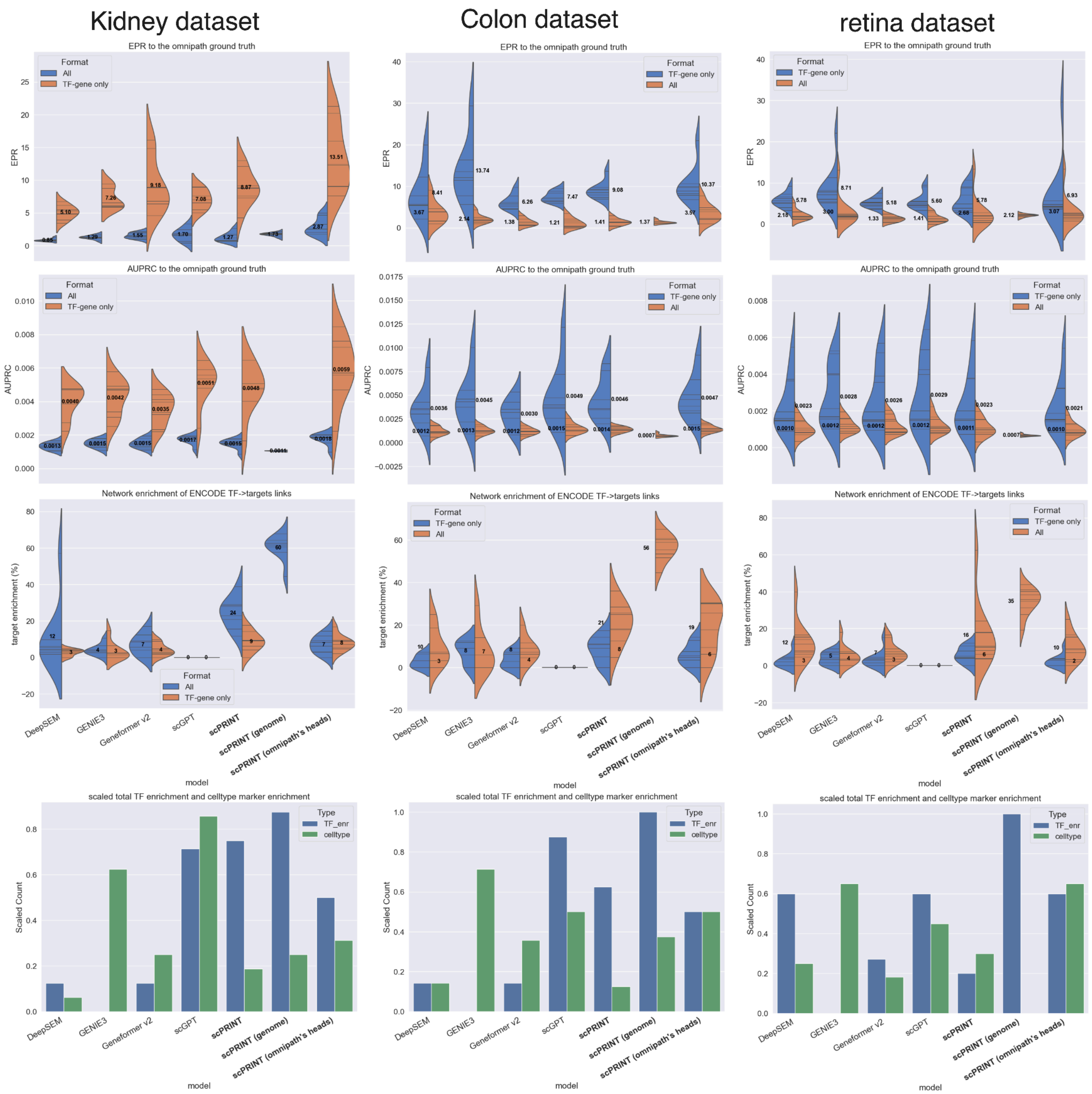

The same plots as in Figures 2B, C, and D, showing the Omnipath and enrichment results per dataset for each of the 3 datasets used. Source data are provided as a Source Data file.

FIG S3: Distribution of connection amongst the three ground truths

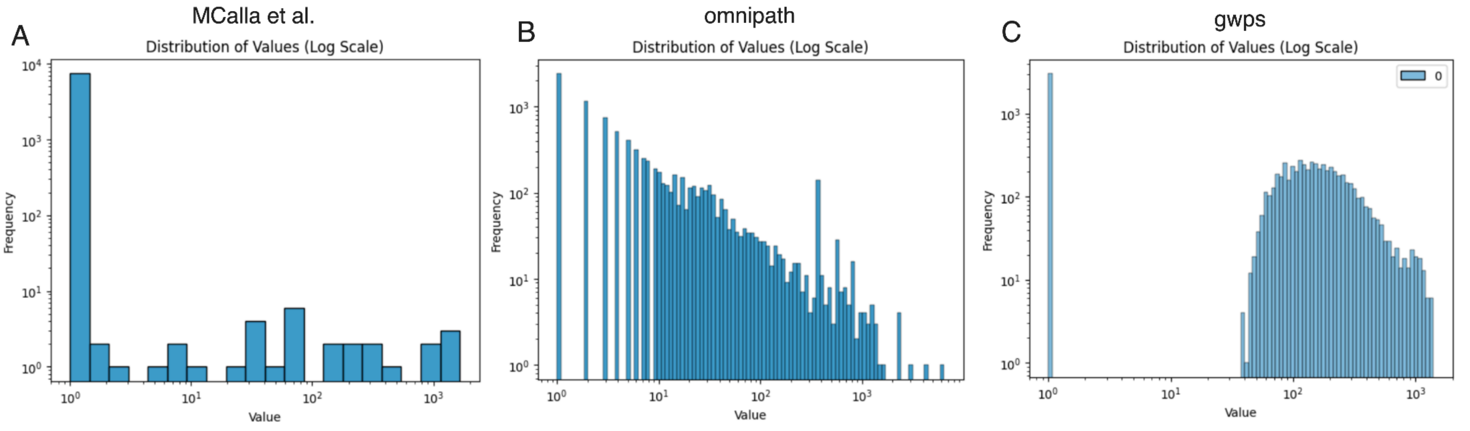

(a) Barplot of the distribution of the number of connections per edge in the MCalla human ground truth network. Most connections are 0, and there is a roughly uniform distribution of connections otherwise. This means most connections belong to the half a dozen most connected edges. (b) Barplot of the distribution of the number of connections per edge in the Omnipath ground truth network. We can see an almost linear relationship on the log-log scale, suggesting a power law distribution. (c) Barplot of the distribution of the number of connections per edge in the genome-wide perturb-seq ground truth network. We can see a very different distribution where only a few genes have little differentially expressed genes post-knock-out, and this trend increases until reaching around 200 connections. Then, it diminishes in what might be a power law. However, some of it is likely caused by the differential expression method and noise in the scRNAseq methodology.

FIG S4: Performance of each GN inference method on predicting the TF-gene only subset of the GWPS ground truth network

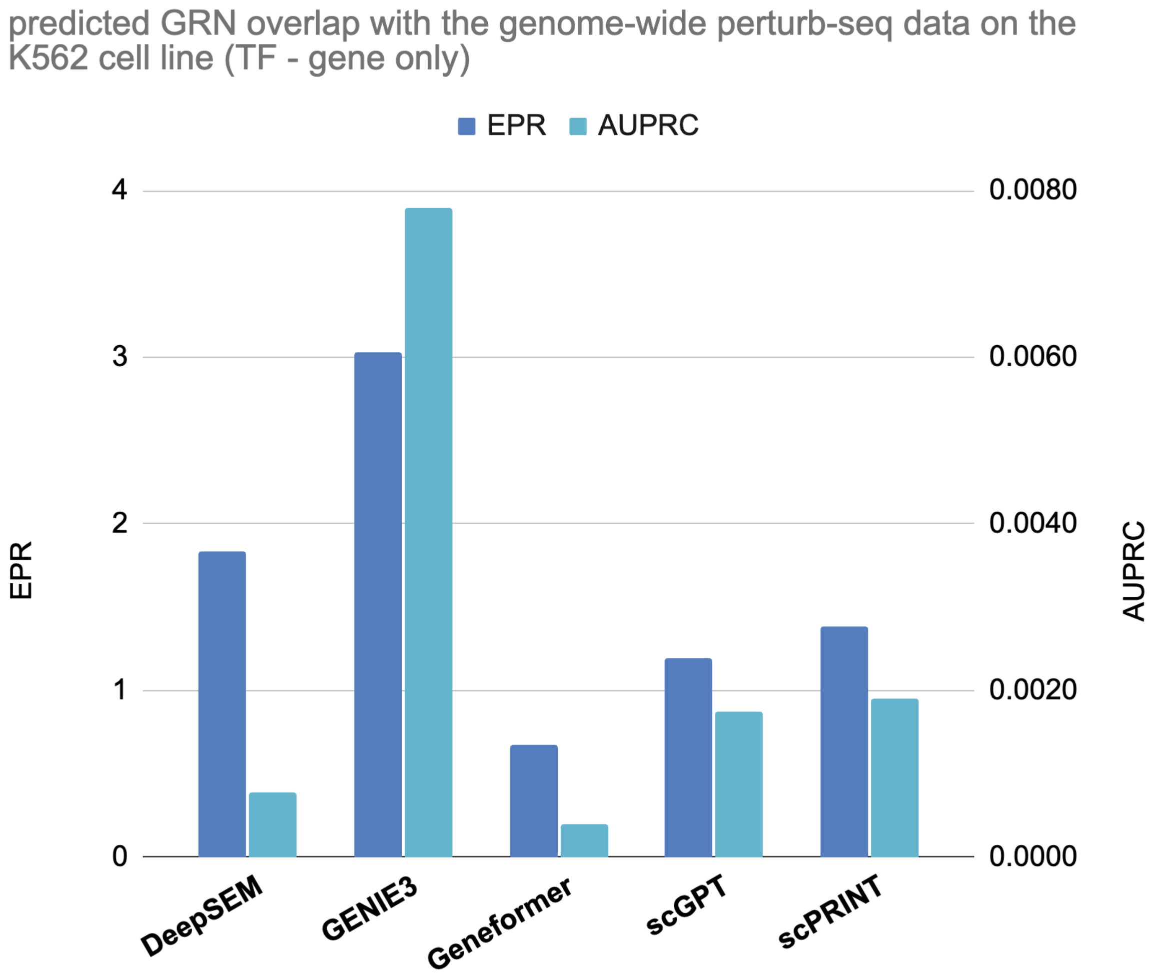

Performances of each model's networks on its overlap with the TF-gene-only subset of the genome-wide perturb seq ground truth. It shows that on this task, most foundation models do not perform well. This could be due to the way their attention matrix is normalized. Source data are provided as a Source Data file.

FIG S5: Full denoising results

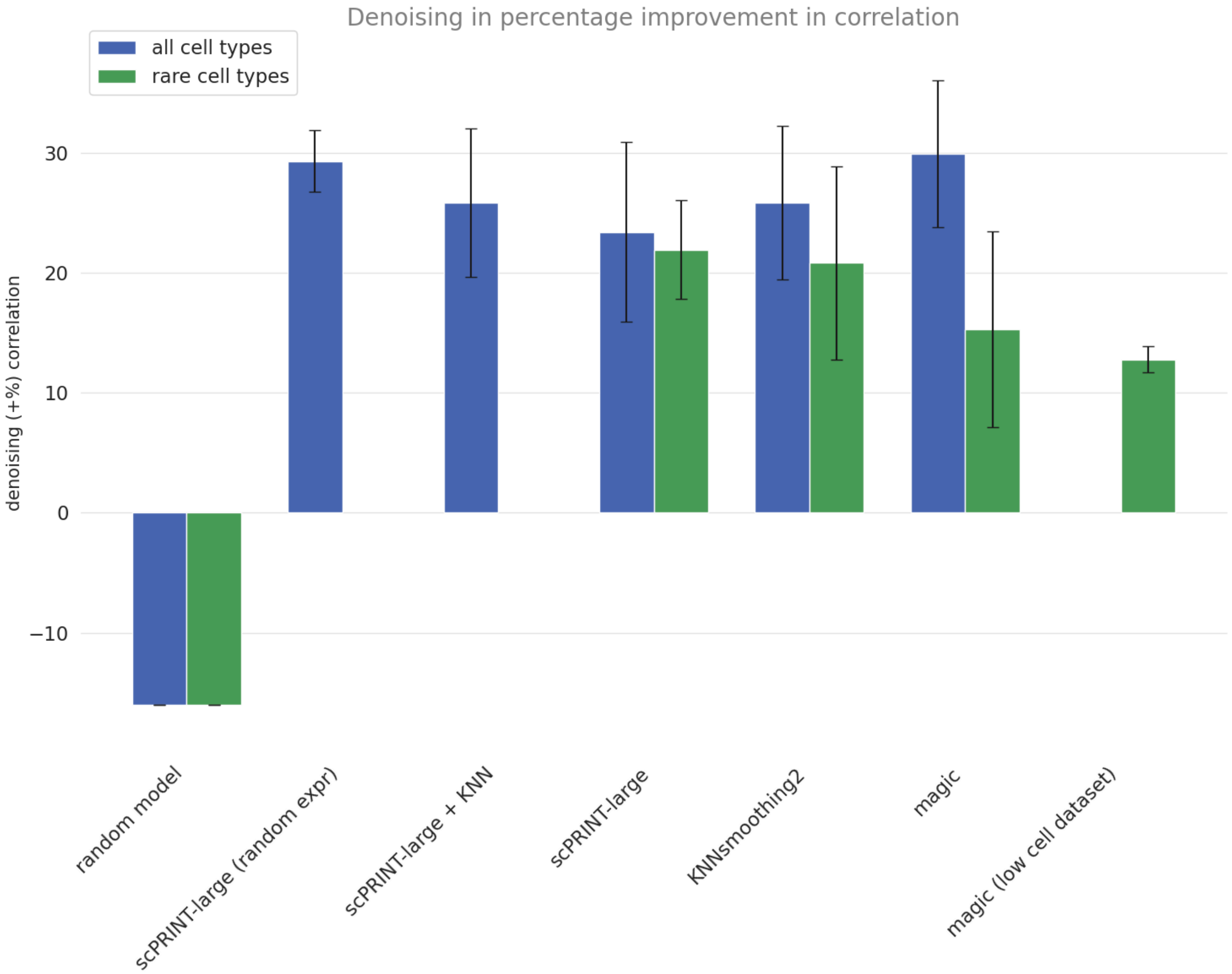

Denoising scores, similar to Figure 4A, but over more tools. “Random model” means a scPRINT model without pre-training. “Random expr” means that scPRINT was using a set of 3000 genes in a similar way as done in pre-training: Taking random expressed genes completed with random unexpressed genes if less than 3000 genes are expressed in the cell. “low cell dataset” means that MAGIC was only using the rare cell population for the dataset as presented in section Denoising validation test of the methods. Source data are provided as a Source Data file.

FIG S6: cell type classification metrics with per-batch split

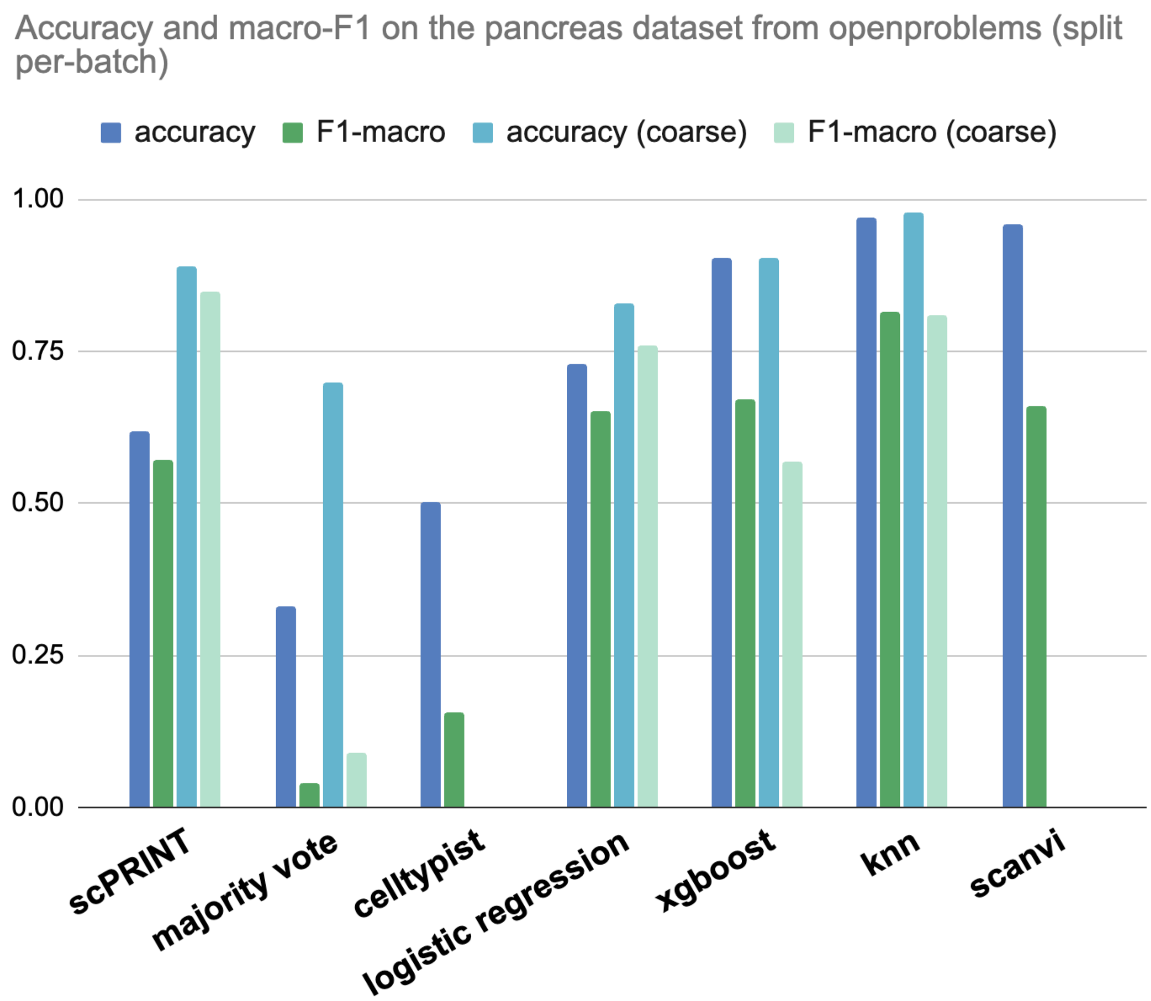

Cell type classification scores over the kidney test dataset of openproblems. Same as Figure 4B, but now the trained methods are trained on a subset of the batches representing roughly 70% of the dataset. The performance is lower in this context, and scPRINT, majority voting, and Celltypist's performance are not changing. Source data are provided as a Source Data file.

FIG S7: Full scIB batch correction scores

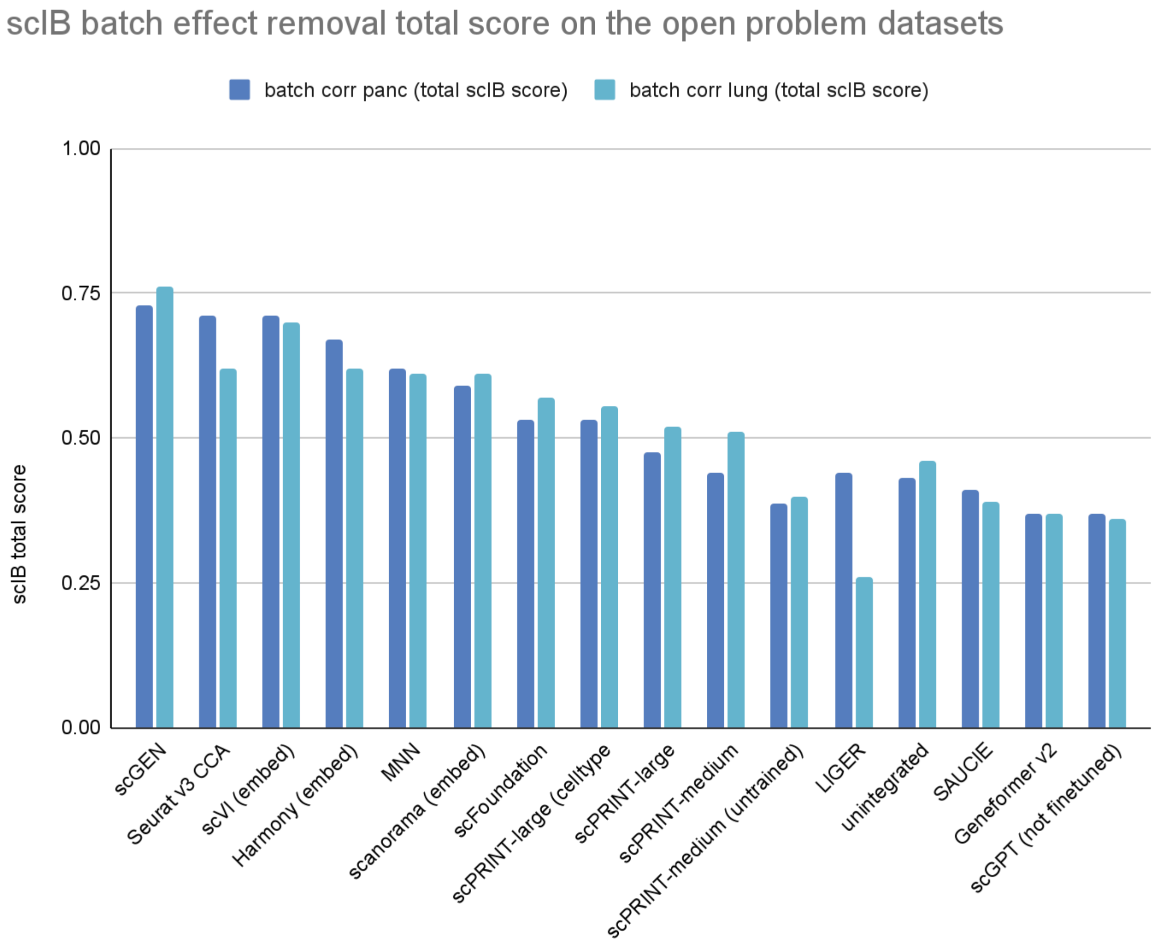

scIB benchmarking scores, averaged for the kidney and lung openproblems test datasets. Same as Figure 4C but over more tools. Cell type logits mean that the logits of the cell type classifier have been used as cell embeddings instead of the cell type embedding itself. Source data are provided as a Source Data file.

FIG S8: Full avgBio scores

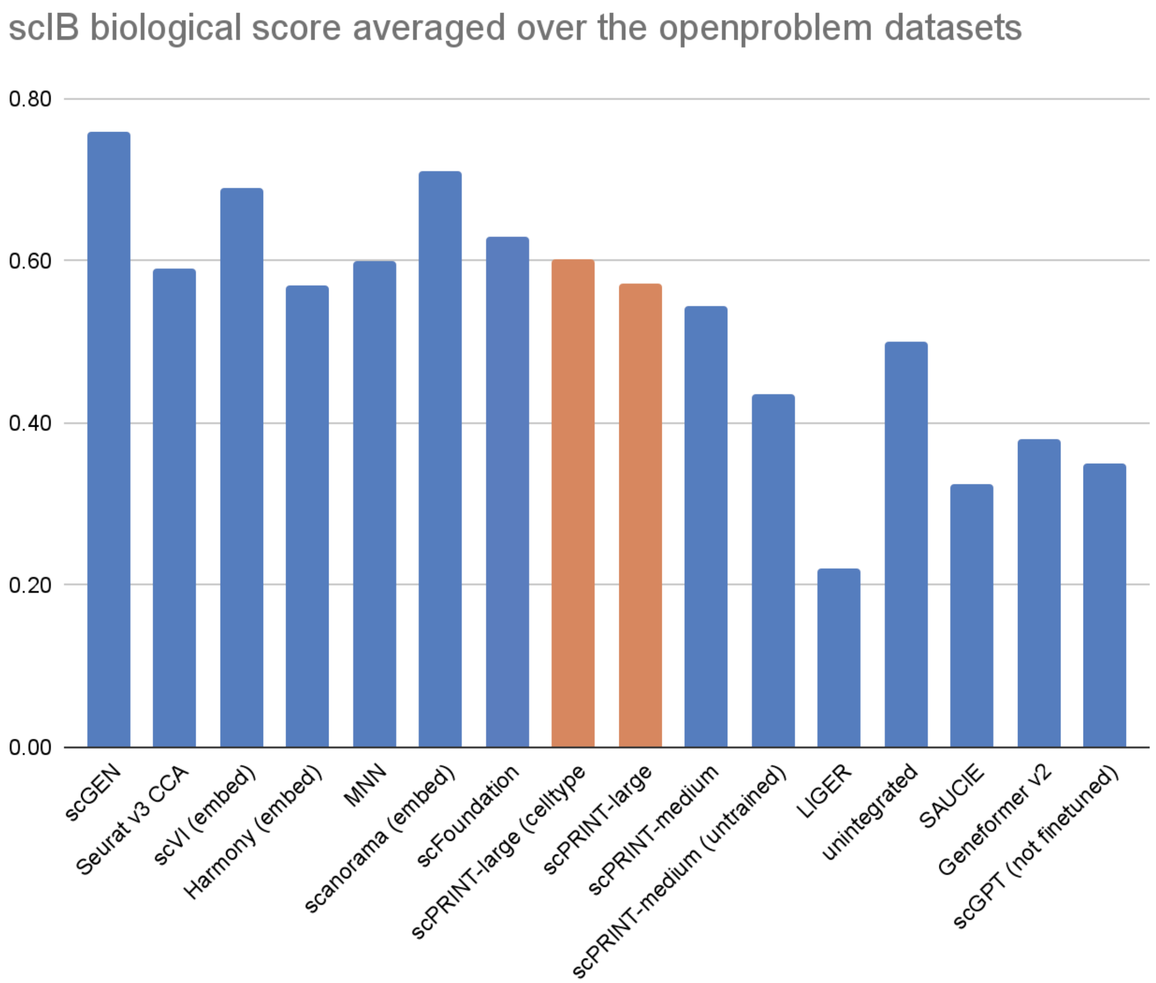

The average Biological score of the scIB benchmark averaged over the kidney and lung openproblems test datasets. Same as Figure 4D but over more tools. Cell type logits mean that the logits of the cell type classifier have been used as cell embeddings instead of the cell type embedding itself. Source data are provided as a Source Data file.

FIG S9: In-depth view of the BPH dataset and its scPRINT-predicted annotations

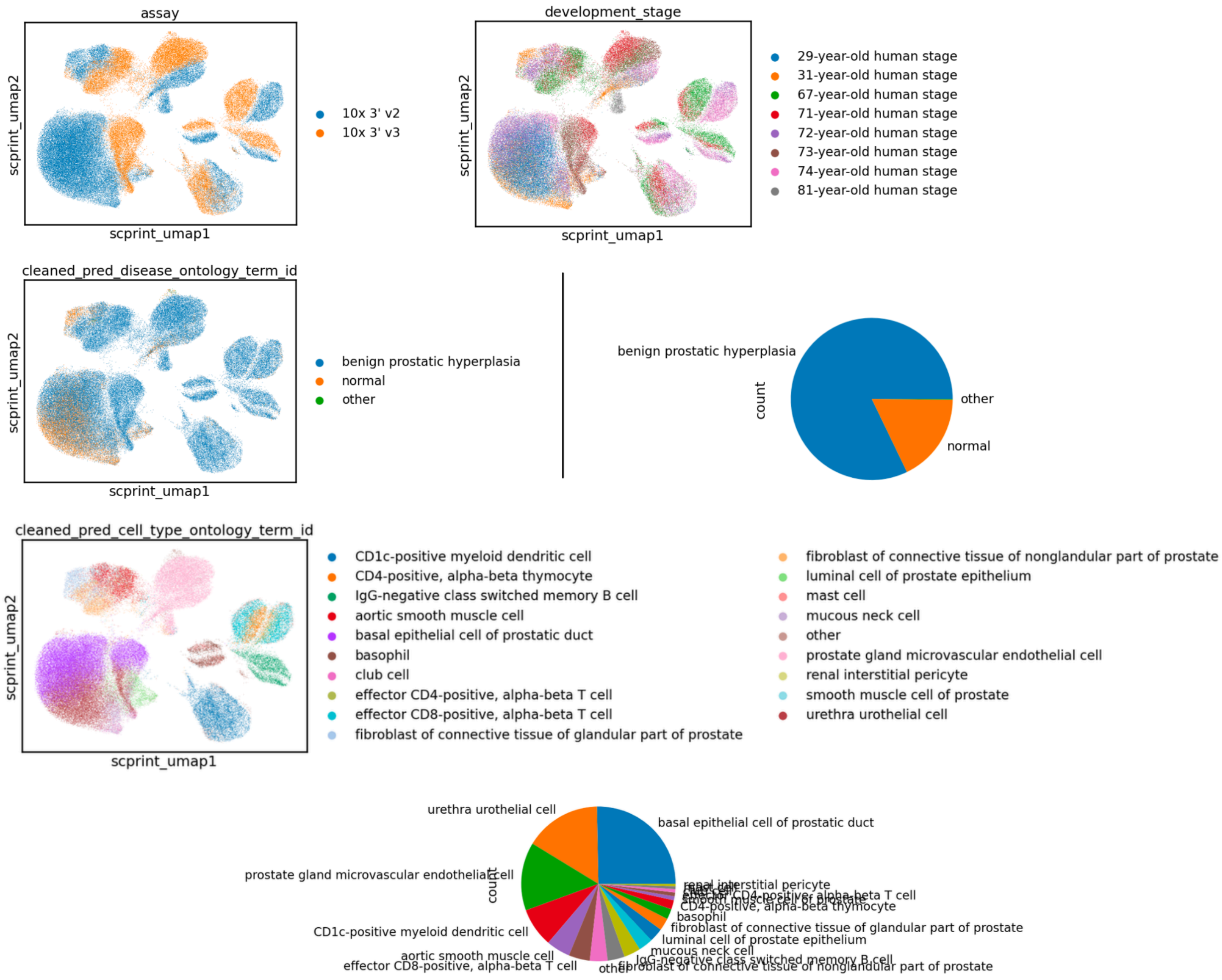

Detailed view of the assay, development stage, scPRINT-predicted diseases, and scPRINT-predicted cell types. Predicted diseases and cell types have been “cleaned” following the strategy presented in Figure 5. We also add pie charts of the relative abundance of each predicted label.

FIG S10: differential expression analysis of the B-cell cluster vs the rest of the cells in the BPH dataset

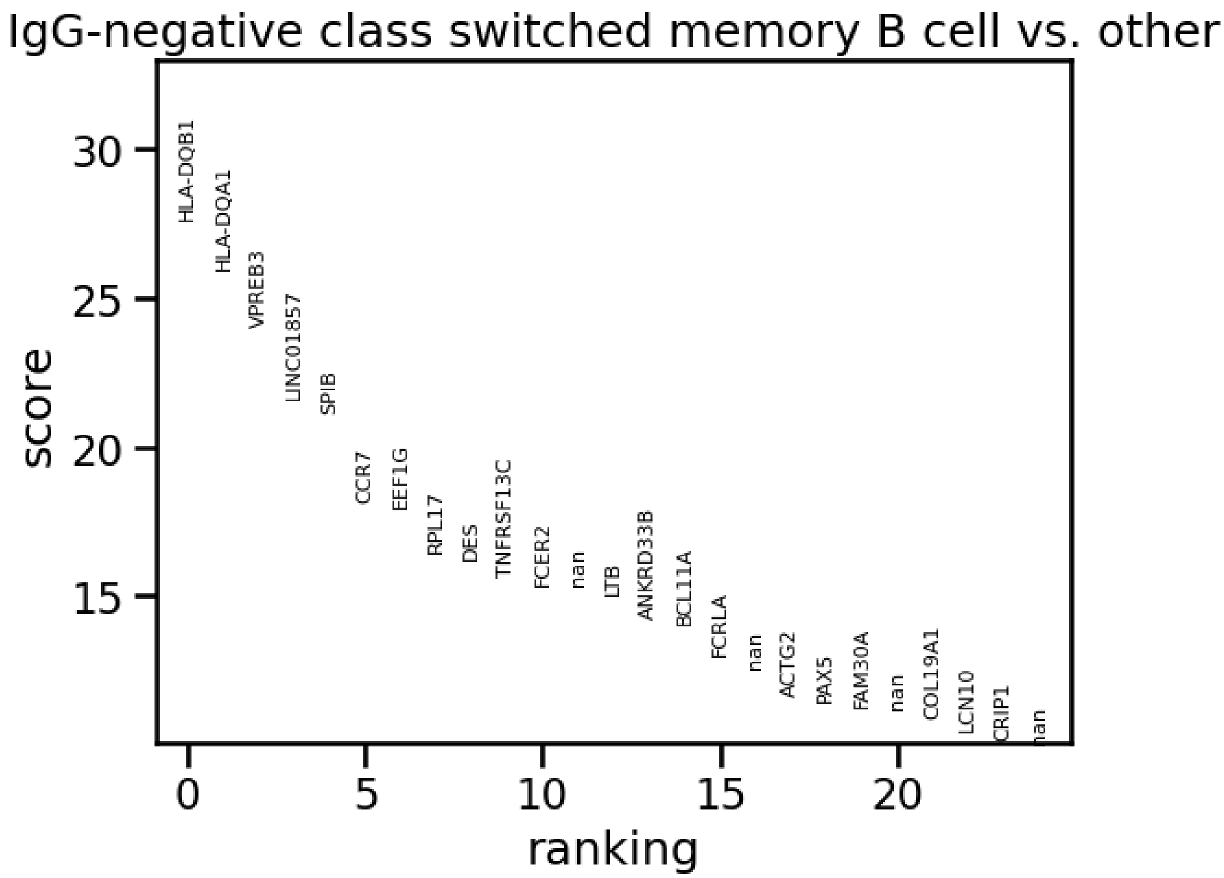

Top genes of the differential expression analysis of the scPRINT inferred B-cell cluster in vs the rest of the cells in the BPH dataset.

FIG S11: gene enrichment comparison in the PAGE4 GN

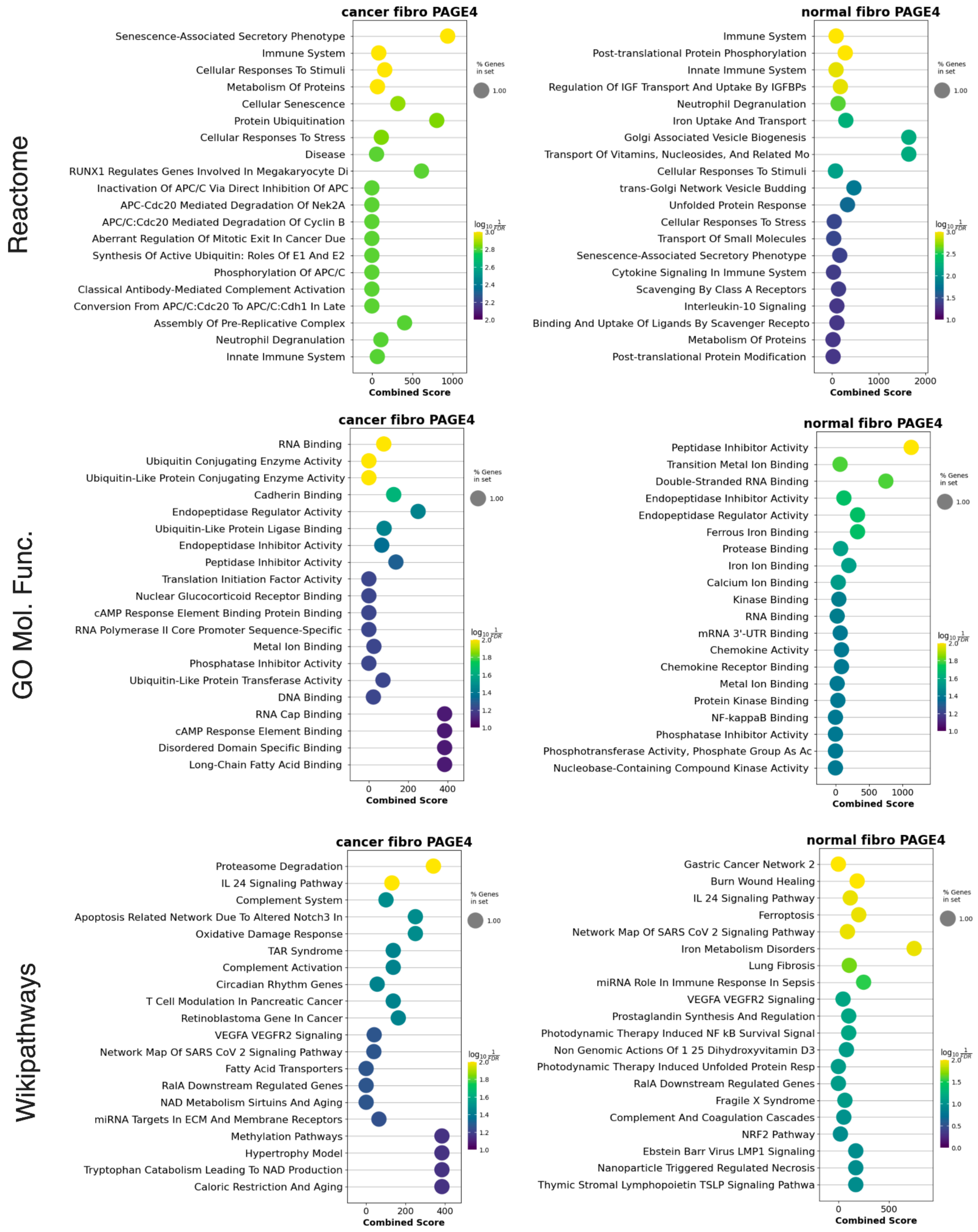

Comparison of the top 20 most enriched terms in Wikipathways, GO molecular function, and Reactome for the 40 most connected genes to PAGE4 in both BPH-associated and normal fibroblast GNs inferred by scPRINT

FIG S12: Gene Network enrichment comparison between the BPH and normal fibroblast on their Louvain communities

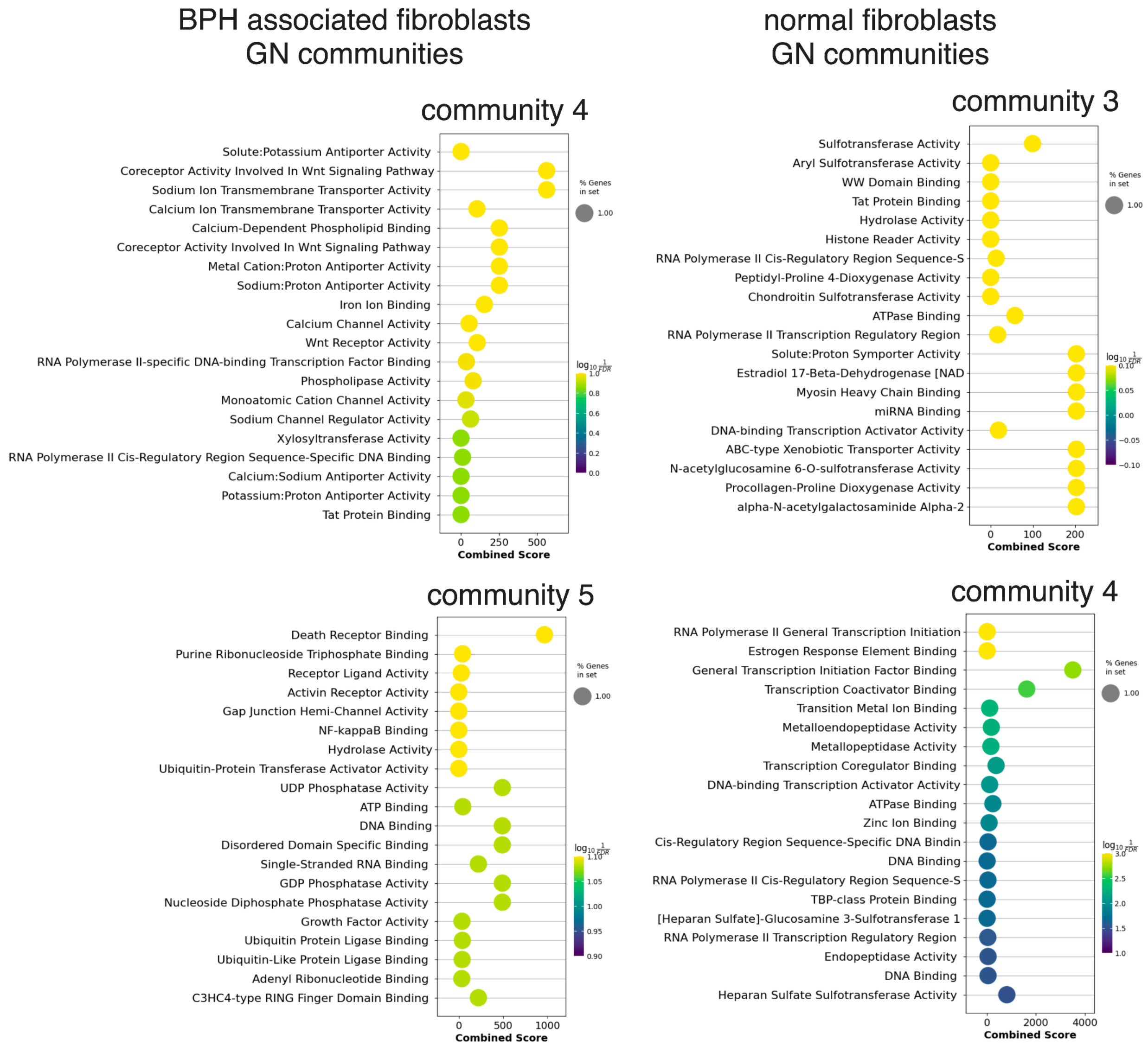

Dotplot of the top 20 GO Molecular function gene sets enriched in the Louvain communities of the BPH and normal fibroblast's Gene Networks.

FIG S13: Graphical Model

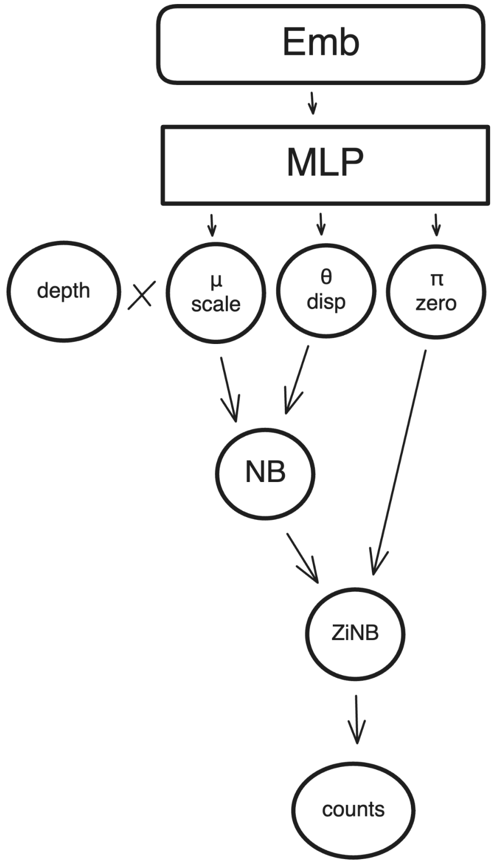

Schematic representation of the zero-inflated negative binomial graphical model of the expression decoder. We generate three values μ, θ, π which are used to model a distribution. We also multiply the μ with the depth (or total count) over the cell.

FIG S14: Hierarchical classifier

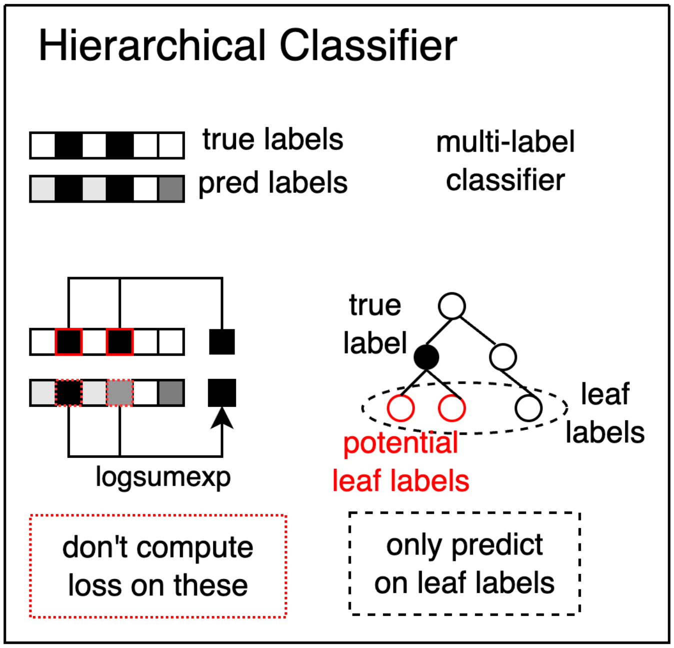

Schematic representation of the hierarchical classifier and its behavior during training. We can train on labels not predicted by the classifier as long as they are parent to one of the predicted labels in the ontological tree.

FIG S15: Detailed representation of the bottleneck learning procedure

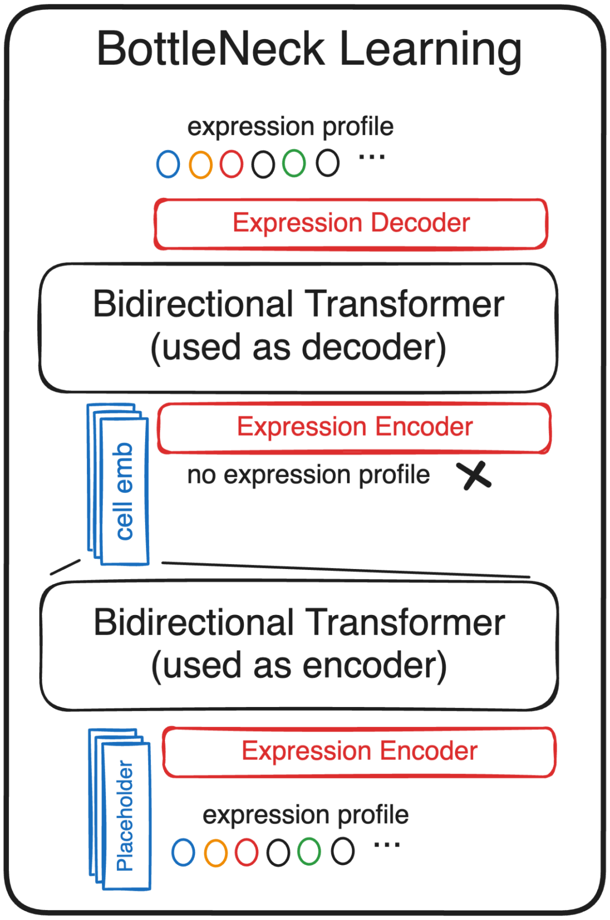

Schematic representation of the bottleneck learning procedure where scPRINT's Bidirectional Transformer Encoder is used both as the "Encoder" and "Decoder" of an auto-encoding (AE) bottleneck learning scheme.

FIG S16: Schematic representation of our dataloader

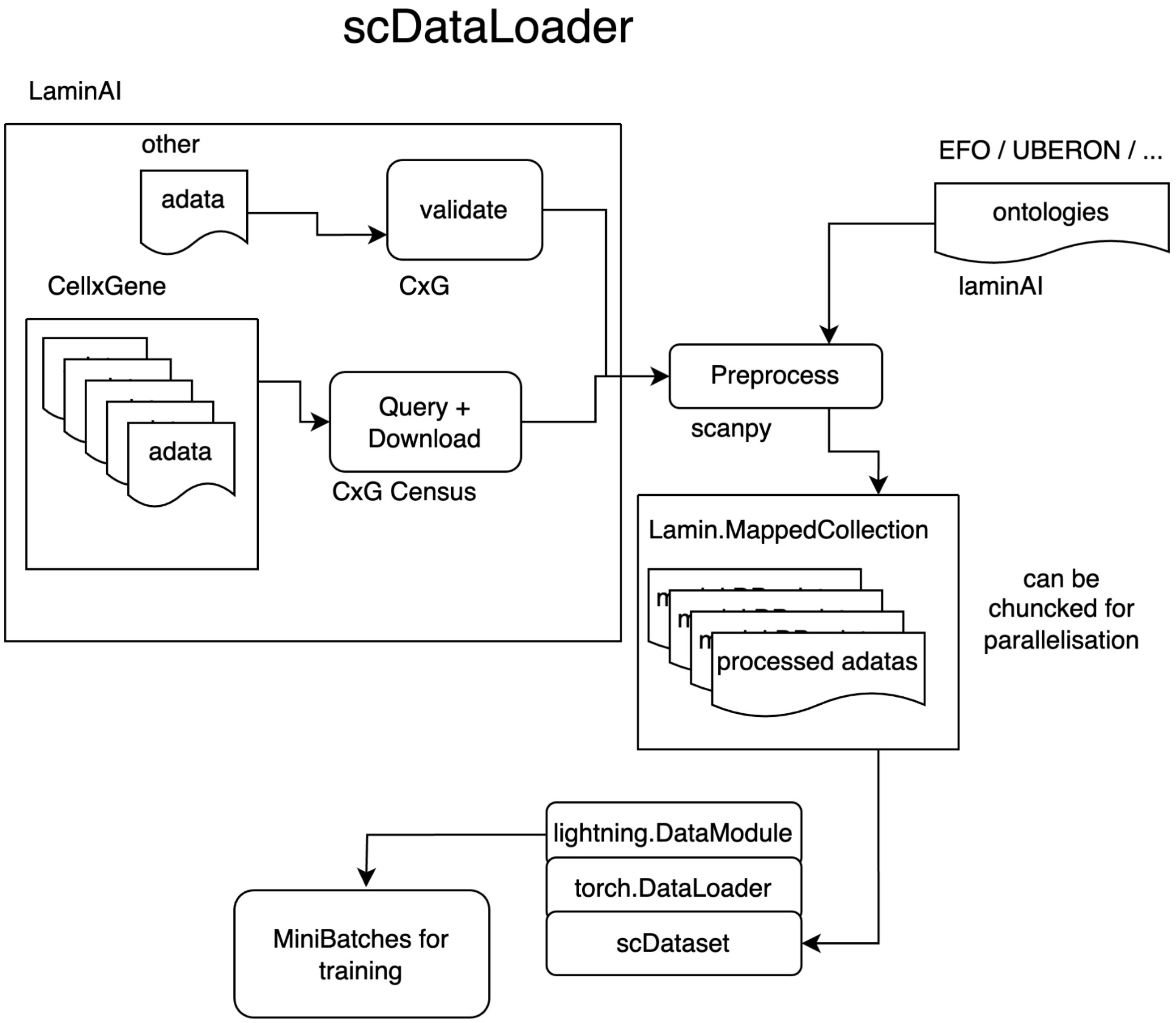

Schematic representation of scDataLoader. Using Lamin.ai, we download and preprocess all cellxgene datasets as AnnDatas. We can also add and validate other expression datasets using lamin.ai. Based on lightning's datamodule framework, torch's dataloaders, our weighted random sampler, and lamin.ai's mapped collection, we can then sample minibatches for pre-training across thousands of datasets and millions of cells with weighted random sampling.
