# Supplementary material for: scPRINT: pre-training on 50 million cells allows robust gene network predictions
Source: Nat Commun. 2025 Apr 16;16:3607. doi: 10.1038/s41467-025-58699-1 (PMC12003772; doi:10.1038/s41467-025-58699-1)
Supplement: Supplementary file 2 — Reporting Summary [file 41467_2025_58699_MOESM2_ESM.pdf]

nature portfolio

Corresponding author(s): Laura Cantini

Last updated by author(s): Jan 22, 2025

Reporting Summary

Nature Portfolio wishes to improve the reproducibility of the work that we publish. This form provides structure for consistency and transparency in reporting. For further information on Nature Portfolio policies, see our Editorial Policies and the Editorial Policy Checklist.

Statistics

For all statistical analyses, confirm that the following items are present in the figure legend, table legend, main text, or Methods section.

n/a	Confirmed	
☐	☒	The exact sample size (n) for each experimental group/condition, given as a discrete number and unit of measurement
☒	☐	A statement on whether measurements were taken from distinct samples or whether the same sample was measured repeatedly
☐	☒	The statistical test(s) used AND whether they are one- or two-sided<br>*Only common tests should be described solely by name; describe more complex techniques in the Methods section.*
☐	☒	A description of all covariates tested
☐	☒	A description of any assumptions or corrections, such as tests of normality and adjustment for multiple comparisons
☐	☒	A full description of the statistical parameters including central tendency (e.g. means) or other basic estimates (e.g. regression coefficient) AND variation (e.g. standard deviation) or associated estimates of uncertainty (e.g. confidence intervals)
☐	☒	For null hypothesis testing, the test statistic (e.g. F, t, r) with confidence intervals, effect sizes, degrees of freedom and P value noted<br>*Give P values as exact values whenever suitable.*
☒	☐	For Bayesian analysis, information on the choice of priors and Markov chain Monte Carlo settings
☒	☐	For hierarchical and complex designs, identification of the appropriate level for tests and full reporting of outcomes
☒	☐	Estimates of effect sizes (e.g. Cohen's d, Pearson's r), indicating how they were calculated

Our web collection on statistics for biologists contains articles on many of the points above.

Software and code

Policy information about availability of computer code

Data collection	Data Collection is performed through the scDataLoader package: https://github.com/jkobject/scDataLoader, the lamin.ai toolkit: https://lamin.ai/, the benGRN: https://github.com/jkobject/benGRN and grnndata: https://github.com/cantinilab/GRnnData packages as well as the omnipath tookit: https://omnipath.readthedocs.io/en/latestapi/omnipath
Data analysis	the benGRN: https://github.com/jkobject/benGRN, GRnnData: https://github.com/cantinilab/GRnnData and scPRINT: https://github.com/jkobject/scPRINT packages are the custom tools used to perform the analysis. A detailed list of open source tools used is available in the requirements.txt file of each packages.

For manuscripts utilizing custom algorithms or software that are central to the research but not yet described in published literature, software must be made available to editors and reviewers. We strongly encourage code deposition in a community repository (e.g. GitHub). See the Nature Portfolio guidelines for submitting code & software for further information.

Data

Policy information about availability of data

All manuscripts must include a data availability statement. This statement should provide the following information, where applicable:

- Accession codes, unique identifiers, or web links for publicly available datasets
- A description of any restrictions on data availability
- For clinical datasets or third party data, please ensure that the statement adheres to our policy

- model weights on: https://huggingface.co/jkobject

- pre-training logs on: https://wandb.ai/ml4ig/scprint_scale/reports/scPRINT-trainings--Vmlldzo4ODIxMjgx?accessToken=80metwx7b08hhourotpskdyaxiflq700xzmzymr6scvkp69agybt79l341tv68hp
- CellxGene datasets: https://cellxgene.cziscience.com/
- All of the other datasets used in this work can be downloaded via the helper scripts on the scPRINT, BenGRN, GRnnData and scDataLoader packages.

Research involving human participants, their data, or biological material

Policy information about studies with human participants or human data. See also policy information about sex, gender (identity/presentation), and sexual orientation and race, ethnicity and racism.

Reporting on sex and gender	sex information was available within datasets and used to train the model. Our BPH result section was a sex specific analysis due to the sex-specificity of the organ. sex/gender disambiguation was left to the appreciation of the cellxgene data curation platform and authors.
Reporting on race, ethnicity, or other socially relevant groupings	ethnicity information was available within many datasets and used to train the model. While we do report classification accuracy on it, ethnicity was not used in other part of our analysis or used for any other association. Methodologies ethnicity reporting is left to the cellxgene data curation platform and authors.
Population characteristics	only disease status was used as part of the metadata available within datasets. No specific associations were made with the disease annotations.
Recruitment	n/a
Ethics oversight	n/a

Note that full information on the approval of the study protocol must also be provided in the manuscript.

Field-specific reporting

Please select the one below that is the best fit for your research. If you are not sure, read the appropriate sections before making your selection.

[x] Life sciences [ ] Behavioural & social sciences [ ] Ecological, evolutionary & environmental sciences

For a reference copy of the document with all sections, see nature.com/documents/nr-reporting-summary-flat.pdf

Life sciences study design

All studies must disclose on these points even when the disclosure is negative.

Sample size	sample sizes were chosen based on amount of publicly available data. We selected as many samples as were available.
Data exclusions	Data exclusion was performed during model pre-training as defined in the methods section. For ground truth samples we excluded some elements as explained in the results and methods section
Replication	All computational experiments and plots are reproducible through python notebooks
Randomization	Most tests were compared against random chance Moreover, models were trained on random splits of the data.
Blinding	blinding of results was not relevant to the study for a few reasons, no experimental data was generated, the project was mostly done by only one researcher. While some notion of blinding might have been possible, they seem very complex to implement in the context of this computational project.

Reporting for specific materials, systems and methods

We require information from authors about some types of materials, experimental systems and methods used in many studies. Here, indicate whether each material, system or method listed is relevant to your study. If you are not sure if a list item applies to your research, read the appropriate section before selecting a response.

Materials & experimental systems

n/a	Involved in the study
☒	☐ Antibodies
☒	☐ Eukaryotic cell lines
☒	☐ Palaeontology and archaeology
☒	☐ Animals and other organisms
☒	☐ Clinical data
☒	☐ Dual use research of concern
☒	☐ Plants

Methods

n/a	Involved in the study
☒	☐ ChIP-seq
☒	☐ Flow cytometry
☒	☐ MRI-based neuroimaging

Plants

Seed stocks	n/a
Novel plant genotypes	n/a
Authentication	n/a
